# Effects of Consumption of Bt-maize (*MON 810*) on the Collembolan *Folsomia candida*, Over Multiple Generations: A Laboratory Study

**DOI:** 10.3390/insects2020243

**Published:** 2011-05-23

**Authors:** Gábor Bakonyi, Anna Dolezsai, Norbert Mátrai, András Székács

**Affiliations:** 1 Szent István University, Department of Zoology and Animal Ecology, 2100 Gödöllő, Páter K. u. 1, Hungary; E-Mails: dolezsaianna@gmail.com (A.D.); matrai.norbert@mkk.szie.hu (N.M.); aszek@nki.hu (A.S.); 2 Plant Protection Institute of the Hungarian Academy of Sciences, Department of Ecotoxicology and Environmental Analysis, 1021 Budapest, Herman O. u. 15., Hungary

**Keywords:** *Folsomia candida*, Bt-maize, life-history, food preference, long-term

## Abstract

The effect of long-term feeding on Bt-maize by collembolans in the laboratory is virtually unestablished. That is why the aim of the present study was to test whether the reproduction, fecal pellet production or food preference of the collembolan *F. candida* is affected when fed on Bt-maize for several consecutive generations. The collembolans were fed with Bt-maize for 0, 6, 16 and 22 months and the number of eggs and fecal pellets were determined. The experiment was repeated seven months later with the same populations. Food preference tests were additionally performed. Significant differences were found in food consumption, egg production and food preference between populations in some cases, but no time-response effect was observed. In conclusion, several generations feeding of *F. candida* on Cry1Ab toxin containing Bt-maize seems not to be harmful to this collembolan species.

## 1. Introduction

Animals are known to be highly important regulators of integrating processes such as decomposition, mineralization, nutrient cycling or CO_2_ production in soil [1]. Soil fauna makes a major contribution to the sequestration and decomposition of soil organic matter. Consequently, possible side-effects of Bt-maize on soil animals may influence the decomposition processes. The input of plant residues into the soil after harvest is as high as 4-9 t ha^−1^ dry material in Hungary [2]. A part of Bt-proteins in soil absorbs and binds irreversibly to soil particles, particularly to the clay fraction and organic matter and the bound proteins are protected against decomposition by soil microorganisms [3]. Cry1Ab toxin in decomposing Bt-maize biomass has been shown to remain biologically active for three years (the longest time studied) [3], for 7 months [4] and at least 8 months [5]. Consequently, long-term presence of the Cry1Ab toxin may have potential side-effects on soil organisms feeding on decomposing Bt-plant material. In turn, collembolans may be exposed to this effect [6].

The long-term effect of Bt-maize cultivation on the soil biota is barely known [6], particularly if the considerable diversity of species [7] is also taken into account. According to Icoz and Stotzky, Bt-plant effects on the soil biota are transient, but possible long-term effects cannot be excluded [8]. Certain studies on this topic were conducted for a relatively long time in the absolute term. Zwahlen *et al.* [5] observed weight loss of the earthworm *Lumbricus terrestris* in a 200-day-long study. The growth of the land snail *Cantareus aspersus* was negatively influenced when feeding on Bt-maize for 88 weeks [9]. However, the time span of the animals was not longer than a single generation even in these experiments.

The multi-generation effects of Bt-plants on soil animals have been reported in very few papers. Knecht and Nentwig [10] found that the number of off-springs and the developmental time of the larvae of two fly species (*Drosophila melanogaster* and *Megaselia scalaris*, respectively) were not influenced when feeding for three or four generations on maize leaves expressing Cry1Ab and Cry3Bb1 toxin. The results were similar when the larvae of the same species were fed for four generations with Bt-wheat [11].

On the whole, no considerable negative effects of Bt-maize on collembolans have been observed in the laboratory [8]. Laboratory experiments were performed usually for one month or shorter. Consequently, these are considered as short-term experiments. Large-scale long-term field experiments were carried out in the framework of the ECOGEN project [12]. Bt-maize effect on collembolan density was found to be neglected [13].

With regard to collembolans, information about long-term effects of Bt-maize on these animals in the laboratory is virtually non-existent. That is why the objective of the present study was to analyze whether the reproduction, fecal pellet production or food preference of the collembolan *F. candida* is affected when fed on Bt-maize for several consecutive generations. Previous experiments conducted with other soil animals showed no Bt-maize effects on earthworms and fly larvae. Considering these results, no effects of several generations long feeding on Bt-maize were expected.

## 2. Materials and Methods

*MON 810* maize strain producing Cry1Ab-toxin (DK-440-BTY) and the isogenic counterpart (DK-440) was used in the experiments. Uniform senescent leaves of similar size and position on the plant were collected at harvest, dried, ground and 1–2 mm long pieces were sieved and thoroughly mixed. Therefore substrate quality did not vary among experiments. Plant material was stored at 4 °C until used as food for collembolan.

C, N and Cry1Ab-toxin content of the leaves were determined by chemical and immunoanalytical methods. Mature leaves were oven dried at 40 °C for 48 hours than homogenized and used for further N and C analysis. The C and N content of plants were determined by Carlo-Erba NA 1500 elemental analyzer [14,15]. The Bt-toxin content was determined by ELISA test [16] using a commercial 96-well microplate format sandwich immunoassay, QuantiPlate® Cry1Ab/Cry1Ac ELISA kit (AP 003, EnviroLogix Inc., Portland, MN, USA).

The collembolan species *F. Candida* was used in the experiments. *F. Candida* is a cosmopolitan species. It can be found almost all over the globe including North America and Europe. It prefers soils with a high level of organic matter, but common in forest and agricultural soils as well [17,18]. Besides, this species is used most common in soil (eco)toxicological tests if a collembolan is the test animal [17].

Trully *et al.* [19] found that 11 laboratory strains of this species fit into two, genetically distinct lineages. The lineages were named as lineage “A” and “B” [18]. The collembolans, used in our studies, belonged to the lineage “B” [20]. The animals were kept under standard conditions during all studies [21]. A stock culture of collembolans was kept and all experiments were conducted in total darkness. Temperature of the incubation cabinet was 20 ± 0.2 °C. *F. Candida* reproduction and fecal pellet production were assessed in the first two sets of experimentation with Bt-maize, while both isogenic and Bt-maize were applied in food preference tests. Collembolans were fed with Bt-maize and baker's yeast ad libitum before the first and between the two sets of experiments on reproduction and fecal pellet production (initial and repeated exposure).

### 2.1. Reproduction and Fecal Pellet Production Experiments

In the first set of experiments testing the effects for the initial exposure of the treated animals to Bt-maize, four populations of collembolan were formed according to their feeding history. All of these four populations consisted of several thousands of collembolans. The four populations were fed with Bt-maize for 0, 6, 16 and 22 months (population symbols were A, B, C and D, respectively) before the first experiment was started. At the start of the first set of experiments 100 individuals per each stock population (A, B, C and D) were randomly chosen. Thereafter the groups containing one hundred individuals were divided by 10 leading to groups involving 10 collembolans. Each group of 10 collembolans was put in standard Petri-dishes with a diameter of 7 cm [21]. In such a way ten replicates were came out in the case of all populations. This set of experiments lasted for four weeks.

During the first set of experiments Bt-maize was given as food *ad libitum*. Baker's yeast (50 mg in each Petri-dish) was also added at the start of the experiment and then once a week to achieve a balanced nutrient composition. The number of eggs and fecal pellets were counted once every week (the method of the fecal pellet counting is described later at the preference test). Upon completion of the first set of experiments on the fourth week, all collembolan belonging to the same population were joined and transported to a new common rearing box and were kept under standard conditions as described before.

The experiment was repeated seven months later using the same collembolan populations. This second set of experiments represented testing the effects for repeated exposure of the treated animals to Bt-maize. Therefore, the feeding duration on Bt-maize prior to the second set of experiments was 13, 23 and 29 months for populations B, C and D, respectively. The experimental conditions (including feeding circumstances) were exactly the same as before (first set of experiments) with the only difference that the population fed with Bt-maize for 0 month (population A) was not the same as in the first set of experiments due to mortality in the latter upon a fungal infection. The second set of experiments lasted for four weeks, as well.

### 2.2. Food Preference Test

Food preference tests were performed individually as described earlier [22]. Briefly, paired choice tests were carried out in Petri dishes with a diameter of 3 cm. One hundred milligrams of ground isogenic or Bt-maize were placed oppositely in the arena. No baker's yeast was offered in this experiment. The number of fecal pellets in a distance of 1 cm around the maize was counted after seven days. This number was regarded as a measure of the food consumption. Individuals were used only once in the experiments. The animals were taken randomly from the populations of the first reproduction and fecal pellet production experiment (from populations A, B, C and D). The number of replication was 40.

Generation time is a key factor when long-term effects are studied. The generation time of *F. candida* is 2–3 weeks [23] at 20 °C. Therefore the number of the generations was calculated dividing the number of the feeding months by 3 (the longest period for generation time given by [23]). The number of generations of *F. candida* in these experiments (6.2 was the longest generation time) was comparable with that of other studies where the generation time varied between 4 and 6 [10,11].

After checking normality one-way ANOVA and post-hoc comparisons with Tukey's test were performed in the case of reproduction and fecal pellet production data. Paired t-test was carried out to analyze food preference [24]. All calculations were carried out using STATISTICA 9.0 program package [25].

## 3. Results

Element content of the maize leafs was as follows: N% 0.27 ± 0.02 and 0.29 ± 0.03 and C% 41.9 ± 4.2 and 40.5 ± 4.5 for isogenic and Bt-maize, respectively. Cry1Ab concentration detected by the QuantiPlate® Cry1Ab/Cry1Ac ELISA kit was 3.45 ± 0.79 μg/g dry leaf material. However, the ELISA kit is based on analytical standards of bacterial Cry1Ab protoxin (molecular mass 131 kDa) and uses antibodies raised against this toxin as immunogen, while *MON 810* maize expresses a preactivated toxin (molecular mass 91 kDa) to which the Cry1Ab-protoxin-specific antibodies show lower affinity (preactivated toxin/protoxin cross-reactivity: 41.2%). As a result, as pointed out earlier [16], Cry1Ab toxin concentrations detected by the ELISA kit are subject to correction with this cross-reactivity. Therefore, calculated Cry1Ab-toxin concentration in the leaf samples used in the present study was 8.38 ± 0.19 μg/g dry leaf material.

**Figure 1:**
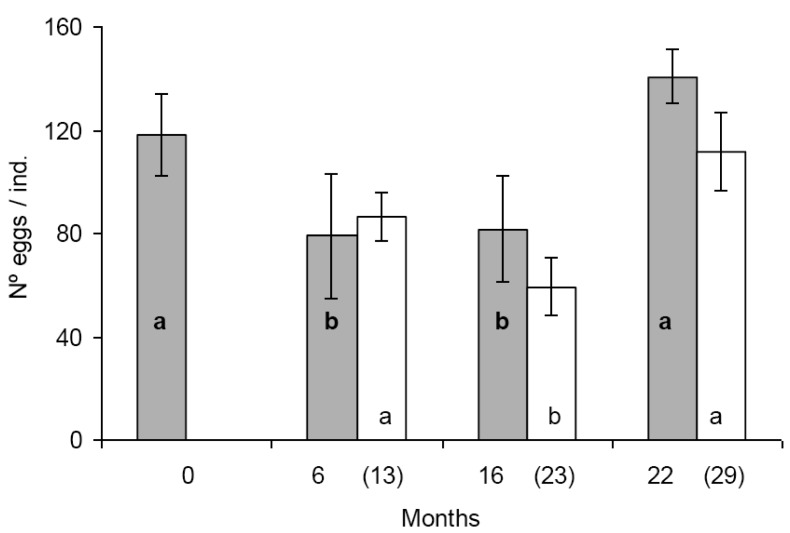
Average number of eggs per individuals (± SD) in *F. candida* populations fed continuously on Bt-maize for various numbers of months. Grey bars represent initial exposure (first set of experiments). White bars represent repeated exposure (second set of experiments). Different lower case letters in the same row indicate significant differences at p < 0.05. Bold lower case letters refers to grey bars, others to white ones. Number of months at the initial and repeated (in parenthesis) exposure are indicated on the x-axis.

**Figure 2:**
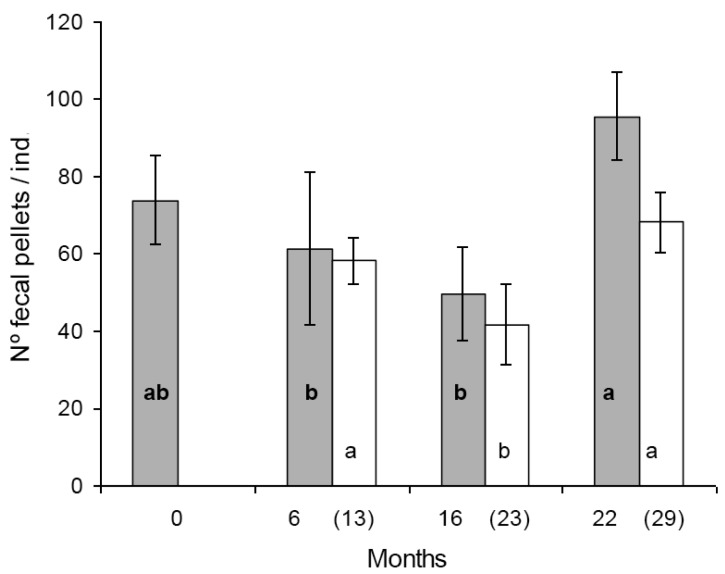
Average number of fecal pellets per individuals (±SD) in *F. candida* populations fed continuously on Bt-maize for various numbers of months. Grey bars represent initial exposure (first set of experiments). White bars represent repeated exposure (second set of experiments). Different lower case letters indicate significant differences at p < 0.05. Bold lower case letters refers to grey bars, others to white ones. Number of months at the initial and repeated (in parenthesis) exposure are indicated on the x-axis.

In the first set of reproduction and fecal pellet production experiments significant differences were found in egg production between some populations. Population A and D produced significantly more eggs than population B and C (Figure 1). The tendency was similar in the second set of experiments, seven months later with the exception that the difference was statistically not significant between population B and D (Figure 1). Similar figure was observed in the case of fecal pellet production. In the first set of experiments population D produced more eggs than population B and C. Population A produced numerically more fecal pellets than population B and C, but the difference was not significant (Figure 2). In the second set of experiments population D produced more fecal pellets than population B and C (Figure 2), however, the difference was not significant between population B and D. No consistent effect of the feeding history on the reproduction and fecal pellet production was observed. Neither egg nor fecal pellet production showed a trend of increase or decrease depending on how long collembolans were fed on Bt-maize before the experiments. Those populations produced most eggs and fecal pellets which were fed on Bt-maize for the shortest (population A) and the longest (population D) duration.

The food selection pattern was also different between the populations (Table 1). Population A and D showed a clear, significant preference for isogenic maize according to the results of the paired t-test (p = 0.001 and p = 0.015, respectively). However, no preference was observed in the case of populations B and C (p = 0.5 and 0.186, respectively).

## 4. Discussion

The Cry1Ab-toxin concentration of DK-440-BTY maize at harvest, 8.38 ± 0.19 μg/g dry leaf material was considerably lower than those found in an earlier study [16]. Nonetheless, it has also been pointed out [26] that toxin content in the leaves near to the soil level are substantially lower than those in leaves at the cob level due to partial necrotization after the R3 phenological stage of maize. Therefore, the Cry1Ab toxin content in the partially desiccated/necrotized bottom leaves were realistic relative to earlier studies [16,26], and the toxin effect was considered as negligible in this experiment.

The main aim of this work was to study whether the reproduction and fecal pellet production of the collembolan *F. candida* is affected when fed on Bt-maize for several consecutive generations. Therefore, only Bt-maize was offered for all populations when reproduction and fecal pellet production were studied. Accordingly, no conclusion can be drawn on the Bt toxin effect itself. In some cases differences in egg and fecal pellet production and feeding preference among populations were high. This is a common phenomenon in similar experiments [10,11]. More interestingly, the rank of the egg and fecal pellet production of the populations remained rather similar if the results of the first and second sets of experiments are compared. Population D had the first, population B the second, population C the third rank (except for the first set of experiments, where the number of eggs was slightly higher in population C than in population B). This observation indicates that collembolans do not adapt to feeding on Bt-maize in the long run. If adaptation would occur, egg and fecal pellet production of the population C should have significantly higher values in the second set of the reproduction and fecal pellet production experiments than those of population B. However, no such difference was found. This phenomenon suggests that these two important population parameters, egg and fecal pellet production are not influenced by long-term feeding of *F. candida* on Bt-maize. A similar observation was made in the case of the flies *Drosophila melanogaster* and *Megaselia scalaris* egg production [10].

Noteworthy association was detected between population parameters and food preference. Populations showing a preference for isogenic maize had higher fecal pellet production and egg number as well. Plant lignin content may play a role in food preference of *F. candida*. Saxena and Stotzky found that four *MON 810* events had higher lignin content than their isogenic counterparts [27]. Corresponding results were found in the case of the MON-00810-6 and SYN-EV 176-9 transgenic maize lines and their isogenic varieties [28] and when three different transgenic maize lines (including one MON810 line, DK647 Bty) were compared with their isogenic counterparts [29]. This may provide an explanation for the preference of the isogenic line by the populations A and D, but not for the observed differences between populations. Consequently food preference mechanisms of collembolan should be studied further. It is possible that individual differences of the food selection behavior could play a role in the observed differences between the populations. This hypothesis needs further investigation.

There are several examples of terrestrial insect populations displaying spatial variation in feeding preferences that suggest local adaptation to plant resources [30]. A similar adaptation is possible for collembolans as well. It is suggested that food preference may help the coexistence of several species in the same soil microhabitat [31,32]. Besides, enough food delays the dispersal of *F. candida* [33]. Therefore, different populations of the same species may show different life-history and food preference patterns. Similarly, Tully and Ferriere [34] have shown high ability for quick adjustment of the reproductive phenotype of *F. candida*.

According to regulations within the European Union, potential adverse effects of Bt-maize on decomposition and nutrient cycling have to be evaluated prior to the introduction of the product to the market [35]. Plant residues are a mixture of relative labile and recalcitrant substrates. Leaves, stalks and roots of maize have considerably different breakdown rates. Stalk decomposition takes at least one year depending on environmental conditions [36,37]. Consequently, the effects should be monitored in the given time frame. The longest time studied was 29 months (6.2 generations) in this research. This duration correctly mimics the field situation. Similar to previous experiments [10,11], no time-response effect was found.

Bt-maize effects on the environment are based on test methods originally developed for pesticides [38]. However, these methods do not fulfill the requirements of the current EU regulations for Bt-maize, because they do not take decomposition and nutrient cycling into consideration, which are key processes in soils. There is a need for more suitable test methods for ecotoxicological assessments of Bt-maize in soil ecosystems [39,40]. Long-term methods are definitely lacking. Comparing ecological attributes of different strains of key factor species exposed to Bt-maize for several generations may be one of the most promising methods in order to discover side-effects of Bt-maize.

## 5. Conclusions

This is the first laboratory study conducted to investigate the potential effects of long-term feeding of the collembolan *F. candida* on Bt-maize. Those populations which showed food preference behavior had higher egg and fecal pellet production, showing that food selection is a key factor in population dynamics. No relationship was found between the studied parameters and feeding history (how long the test animals have been fed on Bt-maize before the experiment) of the collembolan. This finding shows that long-term feeding on maize containing Bt-toxin (Cry1Ab) seems not to be harmful to this collembolan species.

## Figures and Tables

**Table 1:** Fecal pellet numbers (mean ± SD) in food preference tests. p. significance level; N. number of replications.

**Population Symbol**	**Fecal Pellet N° at Isogenic Maize (±SD)**	**Fecal Pellet N° at Bt-maize (±SD)**	**Difference between Fecal Pellet N° at Isogenic and Bt-maize (±SD)**	**P**	**N**
A	9.2 ± 9.8	7.0 ±9.5	2.2 ± 4.1	0.001	40
B	16.8 ±27.5	12.7 ± 13.7	4.1 ± 19.0	0.500	40
C	14.5 ± 14.5	12.9 ± 13.4	1.7 ± 14.7	0.186	40
D	22.3 ± 22.6	16.7 ± 13.6	5.6 ± 13.8	0.015	40
